# Developing Implementation Strategies for the Adoption of the Enhanced Recovery After Surgery (ERAS) Protocols: A Co‐Design Study

**DOI:** 10.1111/hex.70254

**Published:** 2025-04-03

**Authors:** Georgia Tobiano, Joan Carlini, Wendy Chaboyer, Rhea Liang, Keith Addy, Linda Sung, Brigid M. Gillespie

**Affiliations:** ^1^ NHMRC Centre of Research Excellence in Wiser Wound Care, School of Nursing and Midwifery, Griffith University Southport Australia; ^2^ Gold Coast University Hospital, Gold Coast Hospital & Health Service Southport Australia; ^3^ Department of Marketing Griffith University Southport Australia; ^4^ Gold Coast Health Consumer Advisory Group, Gold Coast Hospital & Health Service Southport Australia; ^5^ Faculty of Health Sciences, Bond University Robina Australia; ^6^ Robina Hospital, Gold Coast Hospital & Health Service Robina Australia

**Keywords:** consumers, fast‐track recovery, general implementation theory, participatory action research, surgery

## Abstract

**Background:**

The clinical effectiveness of Enhanced Recovery After Surgery (ERAS) protocols in reducing length of stay and postoperative complications is well established. Yet, the uptake of these protocols remains variable in many healthcare settings.

**Methods:**

We used the Generative Co‐Design Framework for Healthcare Innovation to design and deliver strategies to implement ERAS protocols at one Australian tertiary hospital. Co‐design groups included surgeons, anaesthetists, perioperative and surgical nurses, and health consumers with previous surgery experience. Two workshops with co‐designers were held over 4 months. Textual data derived through workshop artefacts and discussions were analysed inductively. Then, subcategories representing implementation strategies were deductively mapped to the level they primarily target, individual, team and organisation. Finally, using a consensus‐building approach, the top two implementation strategies were ranked across each group.

**Results:**

In total, 36 practitioners across perioperative, ward, surgery and anaesthetics and 4 consumers participated in the co‐design sessions. Through the analysis, 16 implementation strategies were identified, and half of these were aimed at the organisational level. Strategies ranked in the top two commons across all groups of practitioners included reviewing clinical pathways and processes. Consumers believed receiving patient education about ERAS, including its risks and benefits, was essential.

**Conclusion:**

Our findings underscore the intricate nature of coordinating diverse stakeholders in co‐design processes. Despite the challenges this may present, it provides valuable insights and promotes consensus‐driven solutions, ultimately strengthening the implementation of ERAS initiatives.

**Patient or Public Contribution:**

Consumers were involved in the co‐design process and were co‐researchers.

## 1 Introduction

Enhanced Recovery After Surgery (ERAS) protocols have become widely recognised as a strategy used to reduce the physiological stress response of surgery. The number of evidence‐based core components in ERAS protocols varies, depending on the surgical specialty and procedure type, ranging from 10 to 15 components for neurosurgery, to 25–30 for colorectal surgeries [1]. Common components occur pre‐hospital, for example, pre‐operative assessment and information; preoperative, for example, prehabilitation and omission of bowel preparation for selective bowel surgery; intraoperative, for example, opioid‐sparing pain management and maintenance of normothermia; and postoperative, for example, early oral nutrition as tolerated and early removal of urinary catheter [2]. Multiple systematic reviews have found ERAS was associated with decreased hospital length of stay (LOS) without increasing hospital readmission [3, 4, 5]. Other systematic reviews have identified ERAS has the potential to reduce post‐operative complications [6], although its effect on surgical site infection (SSI) has not been established. As further evidence of the benefits of ERAS continues to build, it is evident that ERAS protocols are here to stay and must be incorporated into clinical practice.

The presence of protocols alone is not enough to ensure the implementation of the multitude of components of ERAS. There is still variable uptake of ERAS protocols in areas like Northern Europe [6] and the United States [2]; and in some cases, only some components of ERAS protocols are used [6]. While all 24 ERAS components may not be applicable to all surgeries, there is emerging evidence that many/most should be used. For example, a study of patients undergoing colectomy ERAS protocols across 113 hospitals in the United States showed that higher adherence to ERAS components was associated with earlier recovery, fewer complications and shorter lengths of stay [7]. Complex interventions such as ERAS are challenging to implement [8]. For example, challenges include nurses' lack of knowledge, human resources shortages and lack of cohesion across the multidisciplinary team [9, 10, 11]. Ultimately, approaches to implementing ERAS are just as important as the clinical effectiveness of this evidence‐based intervention.

Researchers have called for a shift from research purely focused on adherence to ERAS protocols (i.e., the intervention) to investigations of the implementation strategies to empower consumers to actively engage in their own care [8, 12]. Rather than treating implementation strategies as a set of techniques to improve compliance, they should be seen as tools that enable consumers to take an active role in shaping and sustaining their care practices. Implementation strategies are techniques used to enhance the adoption or implementation of an intervention; they are the ‘how to’ component of changing practices [13]. Co‐design, a participatory approach characterised by meaningful collaboration, invites consumers and other key stakeholders to work together in developing solutions that address specific care challenges in care models such as ERAS [9, 10, 14, 15]. The co‐design process emphasises the inclusion of diverse perspectives, ensuring that those who will ultimately be affected by the outcome contribute their insights, experiences and needs. In healthcare; co‐design engages patients, caregivers, clinicians and other stakeholder groups to jointly create interventions or systems that best meet their needs [15]. Co‐design fosters shared ownership, enhances relevance and improves the likelihood of successful implementation by creating solutions that are tailored to real‐world contexts.

### 1.1 Implementation Theory

However, recognising the distinct organisational levels of individual/team, unit and organisation is important to the implementation process in evidence‐based practice. The general theory of implementation posits that understanding these levels and their interactions is fundamental to successfully translating research into practice [16]. Components of the theory include *capacity, potential*, *capability* and *contribution,* which can be used to co‐design an implementation plan. *Capacity* refers to the social and structural resources available for the individual/team to enact the new practice [16] (i.e., ERAS), *potential* indicates the social and cognitive resources that allow the strategy to be translated into action, and *capability* suggests the possibility of integrating the strategy into daily clinical routines. Assumptions based on the individual's ability to perform specific activities or tasks related to ERAS reflect *capability*. These three mechanisms influence individual/team contribution, including what individuals will do to implement the strategy in daily practice. *Contribution* is derived from Normalisation Process Theory which comprises four elements: coherence, cognitive participation, collective action and reflexive monitoring [16]. In this study, we focused on the capacity, potential and capability to co‐design an implementation strategy with consumers and healthcare professionals to improve the adoption of ERAS protocols across the preoperative, intraoperative and postoperative periods.

At the individual or team level, strategies can be tailored to address personal motivations and barriers, fostering engagement and accountability [17]. Unit‐level strategies focus on optimising workflows and enhancing team dynamics, ensuring cohesive and collaborative interventions. Meanwhile, organisational‐level strategies must align with broader institutional goals and policies, promoting a unified approach to implementation. To maximise the effectiveness of evidence‐based interventions, it is essential to select and develop strategies that are informed by a comprehensive assessment of the specific context and determinants of implementation gaps [17]. By incorporating these levels and contextual considerations, co‐design enhances the relevance and actionability of interventions, ultimately improving communication and collaboration among stakeholders and supporting more effective and sustainable implementation outcomes.

This study aimed to co‐design an evidence‐based implementation strategy with consumers and healthcare professionals to improve the adoption of ERAS protocols across the preoperative, intraoperative and postoperative periods of surgical care.

## 2 Methods

This co‐design study used a participatory research approach. Co‐design harnesses the expertise and knowledge of end‐users as essential resources in the design process and emphasises equal and reciprocal relationships among all end‐users [15]. The participating hospital (HREC/2023/QGC/100628) and university (2023/396) Human Research Ethics Committees approved this study.

### 2.1 Study Setting and Sample

Co‐designers included health consumers with experience in surgery, perioperative nurses, surgical ward nurses, anaesthetists and surgeons. Healthcare professionals were from one tertiary hospital in Queensland, Australia. Health consumers were members of an advisory group at a nursing research centre of excellence that partnered with the hospital. The inclusion criteria were that a co‐designer identified as one of the groups above and consented to be a member of the co‐design groups. There were no exclusion criteria.

Recruitment of health consumers occurred through an expression of interest process. Health consumers were informed that the next project advisory group meeting would be a co‐design session to gain consumer perspectives on their experiences with ERAS; they were sent information sheets and consent forms prior, allowing them to decide whether to participate or not. For perioperative and surgical ward nurses, educators on the units provided study information to the nurses. Due to the nature of shift work, nurse co‐designers differed across co‐design meetings. Research team members who were anaesthetists and surgeons utilised their networks, providing study information to their colleagues. All participants provided written consent before participating in co‐design meetings.

### 2.2 Co‐Design Process

The study was guided by the *Generative Co‐Design Framework for Healthcare Innovation*, which involves three stages: (1) pre‐design, (2) co‐design and (3) post‐design [18]. This framework offers a practical approach to engaging end‐users seeking to change a specific healthcare process or system. The framework is divided into seven steps within three stages. Supporting Information File 1 provides descriptions of each stage within each step, including the activities undertaken at each step, summarised briefly below.

#### 2.2.1 Pre‐Design

Before initiating co‐design workshops, we had discussions with various hospital staff, including senior leaders and conducted a programme of research to understand current barriers to implementing ERAS. Our preparation for co‐design involved assembling our co‐designers, training three workshop facilitators, developing and testing co‐design activities and organising equipment and meetings.

#### 2.2.2 Co‐Design

Co‐design meetings for consumers and healthcare professionals started the same; an overview of ERAS was provided, goals were set for the meeting, and facilitators ensured that co‐designers had completed consent forms and demographic data collection forms. Specific activities were tailored to the different stakeholder groups involved (see Supplementary Information for activities). For consumers, a storytelling activity was used, with set questions asked of the group to spur discussion of their issues related to ERAS and ideas for implementation. Storytelling was identified as a meaningful activity for consumers based on discussions with the chair of the advisory group, who was also a health consumer, because it allows consumers to share personal experiences, insights and needs in a relatable, human‐centred way [19]. Healthcare professionals participated in co‐design activity worksheets that encouraged them to share ERAS issues and generate implementation ideas. Group discussions occurred across all groups. Additionally, consumer discussions were captured in detailed notes, confirmed by the group, while healthcare professionals' discussions were captured on whiteboards and posters to emphasise priorities. Meeting minutes were documented, meetings were audio recorded, and photos were taken of artefacts generated during the session. Based on co‐designers' needs, consumer meetings occurred virtually, nurse meetings occurred in person, and surgeon and anaesthetist meetings were hybrid. The following four groups all met separately: health consumers, ward nurses, perioperative nurses and medical professionals (surgeons and anaesthetists). To strengthen collaboration, we facilitated structured synthesis sessions where input from different stakeholder groups was presented, discussed and refined. Although participants were not physically co‐located, their contributions were continuously incorporated into a dynamic framework, mirroring an indirect but meaningful dialogue.

#### 2.2.3 Post‐Design

Post‐design involved data analysis and the conduct of a second round of workshops. We conducted a second round of workshops to prioritise the most pertinent implementation strategies identified with healthcare professionals only. Nurses attended in person, and medical professionals attended online. We had to collect consent and demographic data from nurses, as they were different from the nurses present in Workshop 1. Co‐designers were presented with the list of implementation ideas and prioritised their top 2 ideas over two rounds of voting. The second round of workshops included structured activities designed to integrate perspectives from multiple groups. This process allowed participants to reflect on synthesised insights and provide iterative feedback, shaping the final implementation plan.

### 2.3 Data Analysis

For data analysis, audio‐recording transcriptions, activity sheets, photos and meeting notes were uploaded into NVivo for inductive content analysis [20]. This process involved one researcher with extensive experience in qualitative data analysis, including coding and thematic interpretation. They read all materials in their entirety to become immersed. Next, they assigned codes to each line of materials that explained the substance of that line. Initially, health consumers, nurses (perioperative and ward nurses) and medical professionals were handled separately and assigned distinct codes. The codes across co‐design groups were compared, and it became evident that different groups had many similar ideas, suggesting data sufficiency. We considered the broader concept of data sufficiency [21], that is, whether the collected data adequately addressed the research question, accounting for the depth and credibility of the data in answering the study's objectives. Complete data saturation was infeasible, given the exploratory nature of this study. Subsequently, codes across all groups were built into sub‐categories. These sub‐categories were then combined into categories based on commonalities. These final categories represented a list of implementation strategies for ERAS. To ensure rigour, we employed strategies such as reflexivity, peer debriefing and regular discussions with other researchers to validate coding decisions and interpretations.

Following the inductive analysis process, subcategories were deductively mapped to the level they primarily target, individual or team, unit and organisation. While strategies may cross organisational levels, they were classified according to the level they most closely aligned with.

## 3 Results

Each stakeholder group had separate co‐design sessions. Most consumers (60%) were unsure whether they had experienced ERAS as a patient. Table 1 summarises participants' characteristics across all rounds of meetings.

**Table 1:** Sample characteristics of co‐designers.

Characteristic	Perioperative nurses (*n* = 19)	Ward nurses (*n* = 9)	Surgeons/Anaesthetics (*n* = 8)	Consumers (*n* = 5)
Age median (IQR)	38.0 (31.0–47.5)	29.0 (24.5–49.0)	44.0 (41.0–47.0)	49.0 (35.0–62.0)
	*n* (%)			
Sex				
Female	18 (94.7)	8 (88.9)	2 (25.0)	4 (80.0)
Male	0 (0)	1 (11.1)	5 (62.5)	1 (20.0)
Prefer not to answer	1 (5.3)	0 (0)	1 (12.5)	0 (0)
Department				
Perioperative	19 (100.0)	9 (100.0)	5 (62.5)	NA
Surgical			3 (37.5)	
Anaesthesia				
Knowledge level about ERAS protocol*				
Very knowledgeable	0 (0)	0 (0)	0 (0)	0 (0)
Knowledgeable	2 (10.0)	1 (11.1)	5 (62.5)	0 (0)
Neutral	6 (31.6)	2 (22.2)	1 (12.5)	4 (80.0)
Fairly unknowledgeable	6 (31.6)	2 (22.2)	0(0)	1 (20.0)
Very unknowledgeable	5 (26.3)	4 (44.4)	0 (0)	0 (0)
I have experienced ERAS*	4 (21.1)	1 (5.3)	6 (75.0)	1 (20%)
Role* *n* (%)	C/I RN: 11 (57.9) Clinical Facilitator: 3 (15.8) PACU RN: 2 (10.5) Nurse Educator—Perioperative: 1 (5.3) Clinical nurse: 2 (10.5) RN anaesthetic: 3 (15.8) NUM—Perioperative: 1 (5.3)	Surgical ward/unit RN: 9 (100.0)	Consultant surgeon: 5 (62.5) Consultant anaesthetics: 3 (37.5)	NA
Years in current role				NA
< 1 year	2 (10.5)	1 (5.3)	0 (0)	
1–10	11 (57.9)	6 (31.6)	7 (87.5)	
11–20	3 (15.8)	0 (0)	1 (12.5)	
> 20 years	3 (15.8)	2 (10.5)	0 (0)	

Abbreviations: C/I = circulating/instrument, ERAS = Enhanced Recovery After Surgery; IQR = interquartile range, NA = not applicable, NUM = Nurse Unit Manager, PACU = Post‐Anaesthetic Care Unit, RN = Registered Nurse.

* Missing data for surgeons/anaesthetists: 25.0%.

### 3.1 Implementation Strategies

Analysis of data generated from the post‐design stage with healthcare professionals resulted in 16 implementation strategies. These strategies were then classified according to the intended organisational level they were primarily directed towards (Table 2). Half of the strategies (eight) were aimed at the organisational level, while six targeted the unit level, and the remaining two focused on the individual or team levels. However, these strategies overlapped across categories, highlighting the interdependence and high level of interaction required for successful implementation through a whole‐of‐organisation approach.

**Table 2:** Implementation strategies and approaches at the individual, unit and organisational levels.

Level	Strategy	Description	Implementation approach
Individual	Patient education	Implement education to ensure patients are aware of ERAS expectations (e.g., early mobilisation)	Implement workshops where patients/consumers can engage in discussions about their self‐management strategies, e.g., demonstrations and Q&A sessions with HCP.
	Education and awareness	Educate clinicians about what ERAS is and its benefits. Build awareness of upcoming implementation and engage clinicians in the process to heighten buy‐in	Implement a training programme that includes workshops, seminars and hands‐on sessions focused on ERAS protocols.
Unit	Monitor and feedback	Set clear goals and monitor implementation process and intervention compliance against goals. Provide this feedback to clinicians and leaders	Create a real‐time data dashboard that tracks key ERAS metrics (e.g., patient recovery times, complication rates and readmission rates). Share with members of MDT to provide ongoing feedback and foster accountability.
	Appropriate discharge planning alongside ERAS	Enhance discharge process and community support to ensure ERAS patients with shortened length of stay are not re‐admitted	Establish standardised discharge protocols that outline patient education, follow‐up appointments, medication management, etc., so patients are well‐prepared for discharge and have the resources they need for recovery.
	Mid‐level managers take responsibility	Get mid‐level managers to drive ERAS implementation and create a culture of top‐down support. A few keen clinicians cannot make a change	Designate mid‐level managers as ERAS champions in their units, responsible for promoting ERAS principles, providing training and being points of contact for any ERAS‐related questions or concerns.
	Review what is being done well	Review current practices to find ERAS components already done well; focus implementation of ERAS components not done well	Schedule regular meetings where the surgical team can discuss successes and best practices related to ERAS implementation. This will help reinforce positive behaviours and share successful strategies across MDTs.
	Review surgery lists and booking process	Review and change the surgery booking process to facilitate ERAS implementation (e.g., lists need to start on time or earlier and not be overbooked)	Hold weekly or bi‐weekly MDT meetings that include surgeons, anaesthetists, nursing staff and administrative teams to review upcoming surgery lists. Each patient scheduled for surgery is discussed in terms of their suitability for ERAS protocols and adjusted booking processes to ensure adequate time for preoperative preparation (e.g., prehabilitation and patient education).
	Start with one surgery	Implement ERAS with one surgical speciality and then use this success to facilitate further implementation with other specialities	Choose one specific surgery (e.g., hip replacement) to pilot ERAS protocols. Gather data and feedback during the pilot to refine the approach before scaling up to other procedures.
Organisational	Convince leaders of benefits	Convince leaders that ERAS takes time, space and resources to implement. They need to be supportive and focus on different KPIs	Presentations with senior management that highlight outcomes from successful ERAS programmes, i.e., reduced LOS and decreased complication rates, to demonstrate the potential benefits of ERAS implementation.
	Adequate staffing	Hire additional staff to support ERAS (e.g., Administrative, IT, discharge nurses and dedicated staff to oversee ERAS) and ensure good skill mix of staff on shifts to practice ERAS	Perform staffing needs assessment specific to ERAS protocols to identify gaps and advocate for appropriate staffing levels, ensuring that all team members can effectively support ERAS initiatives.
	Focus on exemplars	Investigate places that are doing it well (private hospitals, gynaecologist at the local hospital) and use this to inform the implementation process and/or send staff to observe these practices firsthand to change mindsets	Select a few cases that exemplify successful ERAS outcomes and share these stories with leaders at all levels to inspire and motivate them/their teams to adopt similar practices.
	Review current processes and pathways	Review what surgical pathways are currently practised at the local hospital and clarify the different pathways to: (1) reduce confusion with different terminologies and (2) ensure it is easy to identify a patient on an ERAS pathway	Schedule workshops to map out current surgical processes and care pathways. Involve all stakeholders to identify inefficiencies and opportunities for integrating ERAS principles.
	Enhance teamwork across settings	Use strategies to enhance teamwork across different settings (no examples of strategies provided by the groups)	Implement regular interdisciplinary rounds, including surgical MDT and other key stakeholders, to discuss patient progress and collaboratively address any challenges, fostering teamwork and communication.
	Review pre‐admission and admission﻿ process	Review and change the admission process (e.g., patients do the admission process electronically pre‐admission)	Develop a standardised preadmission checklist that ensures all ERAS‐related interventions (e.g., patient education, preoperative nutrition and prehabilitation) are completed before admission. This checklist should be integrated into the electronic EHR system to prompt clinicians and administrative staff to verify that all necessary preadmission steps are completed efficiently.
	Clinical pathways	Implement pathways that outline steps of ERAS, especially for junior, new and agency staff	Develop evidence‐based and procedure‐specific ERAS clinical pathways for each type of surgery (e.g., HPB, upper GI, colorectal and hernia). Pathways must be standardised across departments and accessible in electronic and printed formats. To support implementation, conduct training sessions for all members of the MDT to ensure consistent adherence to the protocols. Integrate these pathways into the hospital's EHR system with built‐in prompts and reminders to guide clinical decisions and track compliance with ERAS protocols during each phase of patient care.
	Form a special interest group	Form a group that helps make decisions about how to implement ERAS elements in the ERAS protocol and which surgeries ERAS will be implemented for	Create special interest groups in the organisation that focus on specific aspects of ERAS implementation, e.g., pain management or nutrition, allowing for deeper discussions and innovations in those areas.

Abbreviations: EHR = electronic health record, ERAS = enhanced recovery after surgery, HCP = healthcare professional, HPB = hepatobiliary, IT = information technology, LOS = length of stay, MDT = multidisciplinary team.

Analysis of *post‐design* data from health consumers identified one implementation strategy only: patient education. Consumers gave detailed feedback about the information they wanted about ERAS, including risks and benefits, why ERAS was being adopted compared to the usual approach to care, how staff will monitor patients on an ERAS pathway, and what ERAS components entail and how patients can enact them. It was motivating for patients to know that some elements of care may not be required as part of an ERAS pathway (e.g., NG tube insertion). For health consumers, it was critical to tailor ERAS to patients' individual needs, especially for complex patients who might require changes to ERAS components to meet their needs. Including these details in ERAS education was expected. Finally, health consumers highlighted that they wanted the opportunity to communicate during education sessions, to ask further questions and to discuss their options.

The top two common implementation strategies prioritised by each professional group are presented in Table 3.

**Table 3:** Implementation strategy ideas prioritised.

Strategy	Ward nurses	Perioperative nurses	Surgeons and anaesthetists
#1	Adequate staffing	Education and awareness	Start with one surgery
#2	Appropriate discharge planning alongside ERAS and patient education^a^	Clinical pathways	Review current processes and pathways

^a^ Ideas had tied scores.

## 4 Discussion

**Figure 1:**
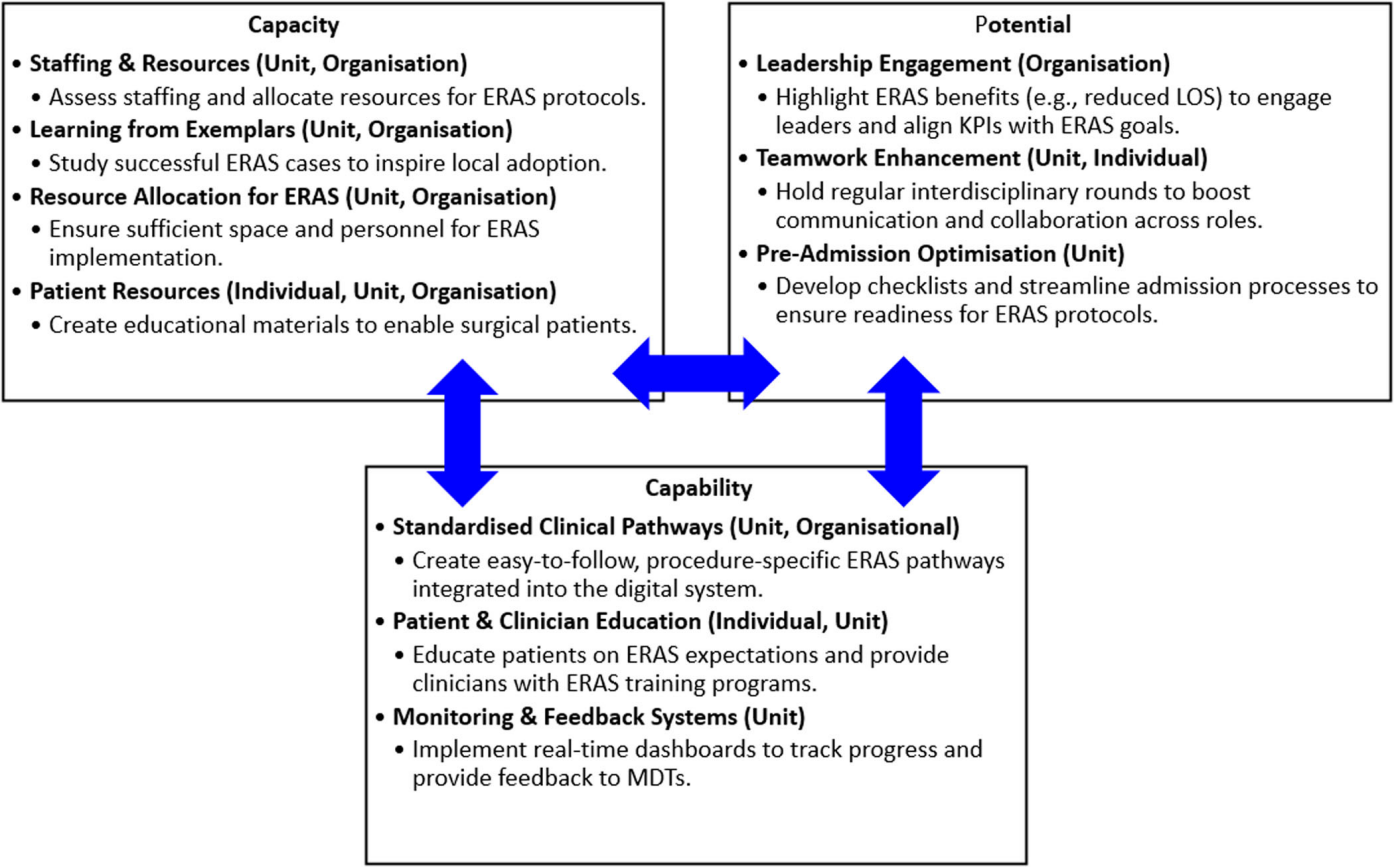
Strategy development for ERAS implementation using the general theory of implementation.

Our aim in using a co‐design approach was to bring about the active involvement of all stakeholders—health professionals and consumers in the development of an effective implementation strategy to bridge the gap in evidence‐based care. This study makes a valuable contribution to the literature by using the Generative Co‐Design Framework [18] to inform the co‐design process. By using this framework, we enabled practitioners and consumers to leverage co‐design artefacts effectively, facilitating the identification and development of implementation strategies. As part of the analysis, we categorised implementation strategies according to individual, individual/team, unit and organisational levels. This allows a comprehensive understanding of the factors that promote or hinder effective ERAS adoption, leading to more tailored and sustainable improvements in patient outcomes. Nevertheless, we recognise a degree of interconnectedness across categories, with some strategies being implemented simultaneously at different levels of the organisation. The findings of the co‐design workshops are discussed through the lens of the concepts of the General Implementation Theory [16] illustrated in Figure 1 to gain a more comprehensive understanding of the context in which the implementation work would be done.

### 4.1 Capacity

*Capacity* is about having available resources such as time, personnel, infrastructure and organisational support [16] for successful ERAS implementation. Fifty percent of the strategies identified fell into the remit of being implemented at the organisational level. Members of our co‐design groups identified the need for adequate staffing to manage increases in additional work processes required to support ERAS implementation, for example, ERAS nurses to coordinate processes. Many others have identified workload issues as a major issue curtailing ERAS implementation [8, 10, 14, 22]. Resource constraints are the most commonly described barrier across different clinical settings with the implementation of clinical practice guidelines [23], delirium prevention [24], the Surgical Safety Checklist [25, 26] and surgical site prevention programmes [27]. Our co‐design participants also identified integrating ERAS with existing processes and care pathways, leading to improvements in the organisation's infrastructure and increasing protocol adherence. For ERAS to be sustainable, healthcare organisations must ensure they have sufficient staffing and access to necessary technology such as electronic dashboards and checklists.

Practitioner co‐designers agreed that support through audit and feedback mechanisms enables MDT to identify gaps and where areas of improvement are needed, as well as enhance collaboration across units and departments within the organisation. Without appropriate resourcing, ERAS may fail to be adopted. There is a clear need to develop knowledge translation capacity and capability‐building programmes to enable experiential and collaborative learning and target participation at all levels [28]. Findings from a recently published scoping review [28] suggest that practice sustainability can only be achieved with the active involvement of senior and middle managers in mediating and supporting practice change and in building staff capacity within their teams. Increasing the capacity of individuals and teams to implement any programme will be influenced by organisational infrastructures and processes [29].

### 4.2 Potential

*Potential* focuses on the readiness and alignment of the organisation to adopt a system or practice change [16] like ERAS protocols. This involves assessing whether HCPs recognise the value of ERAS and whether there is organisational buy‐in for the care pathway. Our surgeon and anaesthetist co‐designers believed that for successful implementation using a phased roll‐out of ERAS, starting with one surgical speciality was necessary to showcase its potential to be scaled up. ERAS protocols trialled with a limited group of patients or specific clinicians before widespread adoption successfully showed local effectiveness [8]. Collecting pilot data to confirm the feasibility of implementing ERAS protocols in the local setting supports their broader application beyond the initial launch phase. The multicomponent nature of ERAS protocols and the increased complexity associated with surgical care coordination in using ERAS has been identified as a barrier to its broader implementation [8]. Starting implementation on a small scale allows implementation processes to be fine‐tuned, can build momentum through the ‘early wins’ of success [8] and may lead to a gradual culture change.

### 4.3 Capability

*Capability* involves the skills, knowledge and expertise required to implement a programme [16] like ERAS effectively. For instance, clinicians must be trained in ERAS protocols, including preoperative, intraoperative and postoperative care pathways. In other literature, education has been identified as the most frequently implemented strategy [28] used in the implementation of practice change programmes. Our HCP co‐designers identified that education was key to building capability in ERAS implementation, while our consumers believed it was important to give patients agency in their care underpinned by the knowledge of the expectations that come with receiving ERAS care. These findings echo those of previous studies examining ERAS implementation [10, 30]. While co‐design group members described education initiatives targeted at the individual level, education strategies need to address diverse stakeholder groups across all levels of the organisation. For instance, the implementation of strategies at the organisational level could include establishing MDT training and learning environments, supporting workplace education and enhancing procedural knowledge and practical skills [14]. These education programmes would also include training on reviewing and improving current clinical pathways to align them with ERAS protocols.

### 4.4 Lessons Learned

The literature often underrepresents the inherent complexities involved in convening co‐designers from various stakeholder groups within healthcare. In our study, we encountered significant logistical challenges in coordinating the presence of all stakeholders at a single event, given the diverse demands on their time and resources. These complexities are compounded by the multifaceted nature of healthcare, where differing priorities, institutional cultures and individual perspectives can create barriers to effective collaboration. Despite the logistical challenges associated with gathering all stakeholders, their commitment to the co‐design process was evident. Participants engaged actively, contributing a range of insights shaped by their respective experiences and areas of expertise. Although the diversity of perspectives initially posed challenges in reaching consensus, it ultimately facilitated a convergence of ideas and solutions. This collaborative effort demonstrated that inclusive dialogue, while complex, can yield innovative and comprehensive outcomes.

### 4.5 Strengths and Limitations

While this study has some strengths, we also acknowledge its limitations. The study was conducted in a single site; therefore, implementation strategies developed through the co‐design process may be context‐specific and may only apply to this local context. To mitigate this, we documented the co‐design process and outcomes comprehensively, highlighting context‐specific elements and identifying implementation strategies applicable at the different levels of the organisation. Despite our best efforts, it was infeasible to have all groups of co‐designers in the same room at the same time. The surgeons and anaesthetists could only meet in the evenings (after completion of elective surgical lists), either online or in person (hybrid meetings). Co‐design meetings with perioperative and ward nurses were conducted opportunistically in person during in‐service meetings. Nevertheless, all participants were highly engaged in the co‐design process; in post‐design meetings, ideas across groups were fed back to the different groups, allowing groups to consider each other's ideas during the prioritisation of implementation strategies. Finally, the research team underwent intensive training in co‐design, by an expert in the field before starting the study. The expert helped develop co‐design activities we used during meetings.

## 5 Conclusions

The integration of evidence‐based implementation strategies is essential for enhancing the effectiveness of ERAS initiatives. However, the implementation remains underrepresented in the existing ERAS literature [10]. The absence of a robust evidence‐based implementation framework may have hindered the effectiveness and sustainability of ERAS practices across various clinical settings. Successful implementation of ERAS protocols relies on a comprehensive understanding of the capacity, potential and capability within healthcare organisations. Our study makes three key contributions: first, it offers a systematic approach for integrating evidence‐based implementation strategies into ERAS protocols, addressing significant gaps in the literature. Second, it provides practical guidance for healthcare practitioners to co‐design ERAS strategies tailored to their specific contexts, facilitating effective stakeholder engagement. Lastly, it identifies critical factors influencing successful ERAS implementation, including the importance of organisational capacity and readiness. Furthermore, our findings highlight the complexities of coordinating diverse stakeholder involvement in co‐design processes. While these challenges may pose difficulties, they ultimately yield valuable insights and foster consensus solutions, enhancing the overall implementation of ERAS initiatives.

## Author Contributions

**Georgia Tobiano:** conceptualisation, methodology, validation, formal analysis, investigation, data curation, writing – original draft, visualisation, project administration. **Brigid M. Gillespie:** conceptualisation, methodology, validation, formal analysis, investigation, resources, data curation, writing – original draft, writing – review and editing, visualisation, supervision, project administration. **Rhea Liang:** validation, data curation, writing – review and editing. **Wendy Chaboyer:** validation, resources, data curation, writing – review and editing. **Keith Addy:** validation, data curation, writing – review and editing. **Joan Carlini:** validation, formal analysis, data curation, writing – review and editing, visualisation. **Linda Sung:** data curation, writing – review and editing.

## Ethics Statement

The participating hospital (HREC/2023/QGC/100628) and university (2023/396) Human Research Ethics Committees approved this study.

## Conflicts of Interest

The authors declare no conflicts of interest.

## Supporting information

supmat.docx.

## Data Availability

The datasets generated and/or analysed during the current study are not publicly available due to ethical and privacy restrictions but are available from the corresponding author upon reasonable request.
