# Supplementary material for: Developing Implementation Strategies for the Adoption of the Enhanced Recovery After Surgery (ERAS) Protocols: A Co‐Design Study
Source: Health Expect. 2025 Apr 3;28(2):e70254. doi: 10.1111/hex.70254 (PMC11968776; doi:10.1111/hex.70254)
Supplement: Supplementary file 1 — supmat.docx. [file HEX-28-e70254-s001.docx]

**SUPPLEMENTARY FILES**

**Supplementary file 1:** Description of stages and steps in the Generative Co-Design Framework for Healthcare Innovation ^18^, and how these were actioned.

| **Stage** | **Step** | **Description** | **Actions undertaken in this study** |
| --- | --- | --- | --- |
| PRE-DESIGN | 1. Contextual inquiry | Aims to understand end-users’ challenges and current practices. | Exploratory program of research conducted:   - Australia-wide survey to understand current ERAS practices, and barriers and facilitators. - Australia-wide interviews to explore current ERAS practices, barriers, and facilitators in more depth. - Systematic review of reviews on ERAS effectiveness for outcomes like hospital re-admission. - Informal conversations with key people at the study site to understand current ERAS practices. |
|  | 2. Preparation & training | End-users and facilitators become acquainted with the project and begin to build rapport. Development and distribution of informational materials related to the project and recruit co-design session facilitators. Thorough preparation for co-design meetings. | - Study materials (flyers, information and consent forms) were distributed to health consumers, nurse educators, and our surgeon and anaesthetist networks to facilitate the recruitment of co-designers. - Rapport was already built with health consumers, surgeons and anaesthetist co-designers, as they were known by research team members. Due to the nature of nursing shift work, we could not build rapport with nurse co-designers before the day of the workshop; however, nurse co-designers knew each other through facilitated interactions. - Three facilitators (GT, JC, BMG) underwent intensive training in co-design workshops. - Co-design activities were developed with the assistance of an expert and trialled with a group of academics and students with health backgrounds. - All equipment (e.g. audio-recorders) were gathered and tested before the day. Food was organised for all in-person sessions. - Rooms were booked for in-person meetings; online meetings were arranged. The research team worked with consumer, surgeon, and anaesthetist co-designers to identify the best time for meetings, to maximise attendance. For nurse co-designers the educator identified the best time for meetings. |
| CO-DESIGN | 3. Framing the issue | Establish a mutual understanding of the lived experiences and challenges faced by end-users as well as a shared vision for the work. | In healthcare professional co-design meeting 1, the following occurred:   - Brief PowerPoint defining ERAS and sharing some benefits presented. - Goal for co-design meetings presented (address the question “how might ERAS be implemented”?), and ground rules for meetings provided. - Undertake the activity “hopes, legends, fears/myths and taboos” to understand common experiences and challenges faced in implementing ERAS.   In consumer co-design meeting 1, the following occurred:   - Brief PowerPoint defining ERAS and sharing some benefits presented. - Goal for co-design meetings presented (address the question “what might we need to do for patients if we implement ERAS”?), and ground rules for meetings provided. - A ‘persona’ was created for a person (Alex) experiencing ERAS at their local hospital, and the facilitator used story-telling to explain Alex’s journey through ERAS. The facilitator paused at three times points (pre-operation, intra-operation, and post-operation). When paused, the facilitator asked the consumers “How do you feel about the care Alex is receiving”, which allowed the group to share their lived experiences and any challenges they foresaw. |
|  | 4. Generative design | Considers the explicit and latent needs of stakeholders and end-users relative to the causes of the problems and possible solutions based on stakeholders’ and end-users’ expertise and experience. | In healthcare professional co-design meeting 1, the following occurred:   - Undertake the activity “start, stop, continue” to generate ideas for implementation strategies. Healthcare professionals were instructed to think about current ways of working, plus the fears and taboos identified in the previous activity, and then identify what needs to start, stop and continue at each level of the organisation (micro, meso and macro levels).   In consumer co-design meeting 1, the following occurred:   - As mentioned above, the facilitator used story-telling to explain Alex’s journey through ERAS. The facilitator paused at three times points (pre-operation, intra-operation, and post-operation). When paused, the facilitator also asked consumers “*If you were in a similar situation to Alex, what would like to know and what would you like to be done?”*, which allowed the group to share their ideas for solutions to implementing ERAS. |
|  | 5. Sharing ideas | End-users to share ideas encountered in the current state. The ideas are intended to build an understanding of the need for clinical change. The research team should pay attention to similarities and differences created by different groups, points of emphasis by participants, and stated priorities for the future state of healthcare. | In healthcare professional co-design meeting 1, the following occurred:   - Co-designers first completed activities silently and independently (“hopes, legends, fears/myths and taboos” and “start, stop, continue”), and then to encourage sharing of ideas, group discussion was facilitated to share ideas generated during the activities, recorded on a whiteboard or butcher’s paper. These documentations allowed us to collate the ideas and see similarities and differences. Additionally, audio-recorded discussions captured points of emphasis and priorities.   In consumer co-design meeting 1, the following occurred:   - The facilitator coordinated group discussions allowing consumers to build on each other’s ideas. One research team member kept detailed notes of these discussions during the meeting. At the end of the meeting, they summarised the similarities and differences shared by the group and the priorities the group identified. The group then had the opportunity to confirm this summary and add to it. |
| POST-DESIGN | 6. Data analysis | The research team sorts data to identify categories and engages in the process of checking in with stakeholders and end-users to ensure the findings reflect stakeholders’ and end-users’ views. Data analysis aims to capture the most pertinent and significant ideas, which will then be used to form the basis of healthcare innovation. | - Audio-recordings of meetings, activity sheets, and photos of whiteboards/ butcher’s paper were uploaded into NVivo Version 20, and inductive content analysis was used to identify categories of implementation strategies in the data.   In healthcare professional post-design meeting 1, the following occurred:   - We returned to the co-designers with the findings of our analysis and presented the list of implementation strategies from across all groups and asked them to prioritise ideas. Over two rounds of voting each group identified the top 2 implementation strategies:   - For nurses, in Round 1 they voted on a paper sheet, their scores were calculated, and then the top-scoring items were recorded on butcher’s paper. In Round 2, they voted again on the narrower list of high-scoring ideas. They were given an activity workshop to write down why they selected these top 2 ideas, and a group discussion was facilitated and recorded.   - For surgeons and anaesthetists, all attended online. Mentimeter was used to vote for Round 1 (full list of implementation ideas) and Round 2 (narrower list of top-scoring ideas from Round 1). Finally, in Mentimeter, co-designers were asked to provide open-ended responses as to why they selected these top 2 ideas, and group discussion was facilitated and recorded. |
|  | 7. Requirements for translation | The research team uses the categories derived from co-design to decide on priorities for the intervention, plans the intervention based on what can reasonably be achieved, and finally closes the loop with co-design participants and stakeholders to identify plans for moving forward with the intervention. | - Our research team considered the prioritised implementation ideas (conceptual at this stage), and determined the actions needed for each implementation strategy identified. We considered what was feasible and what was not. We also engaged our wider stakeholders (i.e. Directors, NUMs, educators etc.) – to discuss what can reasonably be achieved from the ideas prioritised. - Will inform co-designers of the final findings. |

*Abbreviations:* ERAS=enhanced recovery after surgery; NUM=nurse unit manager,
